# Understanding Inter-Individual Variability in Monoclonal Antibody Disposition

**DOI:** 10.3390/antib8040056

**Published:** 2019-12-04

**Authors:** Veena A. Thomas, Joseph P. Balthasar

**Affiliations:** Department of Pharmaceutical Sciences, School of Pharmacy and Pharmaceutical Sciences, University at Buffalo, The State University of New York, 452 Kapoor Hall, Buffalo, NY 14214, USA; vthoma03@amgen.com

**Keywords:** inter-subject variability, pharmacokinetics, monoclonal antibodies, disease, co-administered drugs

## Abstract

Monoclonal antibodies (mAbs) are currently the largest and most dominant class of therapeutic proteins. Inter-individual variability has been observed for several mAbs; however, an understanding of the underlying mechanisms and factors contributing to inter-subject differences in mAb disposition is still lacking. In this review, we analyze the mechanisms of antibody disposition and the putative mechanistic determinants of inter-individual variability. Results from in vitro, preclinical, and clinical studies were reviewed evaluate the role of the neonatal Fc receptor and Fc gamma receptors (expression and polymorphism), target properties (expression, shedding, turnover, internalization, heterogeneity, polymorphism), and the influence of anti-drug antibodies. Particular attention is given to the influence of co-administered drugs and disease, and to the physiological relevance of covariates identified by population pharmacokinetic modeling, as determinants of variability in mAb pharmacokinetics.

## 1. Introduction

Monoclonal antibodies (mAb) are generally found to exhibit desirable pharmacokinetic (PK) characteristics such as slow clearance and long biological half-lives; however, significant inter-individual variability (IIV) in PK is often noted. Anthropometric variables (weight, body surface area), demographic variables (age, gender, and race), anti-drug antibodies (ADA), serum albumin, dose, co-administered drugs, and co-morbidities are often considered as covariates in clinical population modeling of antibody PK. Despite inclusion of these covariates, much of the IIV remains to be explained. Relatively few dedicated studies have been undertaken to examine determinants of variability in mAb PK, and little effort has been placed on the identification of mechanistic biomarkers of IIV. In this review, we discuss (a) mechanisms of mAb pharmacokinetics, (b) variability in determinants of mAb disposition, (c) common covariates identified through population PK modeling, and (d) factors contributing to IIV. We assess and analyze evidence from in vitro, pre-clinical, and clinical studies to understand the sources of IIV and their implications on mAb disposition.

## 2. Mechanisms Influencing Antibody Pharmacokinetics

### 2.1. Mechanisms of Antibody Absorption

Intravenous (IV) administration is commonly employed for mAb dosing, but there is substantial interest in extravascular dosing for mAb. The subcutaneous (SC) route is a convenient option; however, complex PK, site-related proteolytic catabolism, incomplete bioavailability (between 20–95%) [1], injection volume restrictions (limited to 1–2 mL), and concerns for increased immunogenicity complicate the pursuit of this route. Adalimumab, canakinumab, denosumab, golimumab, omalizumab, rituximab, tocilizumab, trastuzumab, and ustekinumab are currently approved for SC administration. In clinical investigations of therapeutic mAb, the IIV observed in SC bioavailability and the rate constant of SC absorption is typically high, with coefficients of variation ranging from 40% to 53% [2]. Determinants of convective transport, the site (anatomical) of injection, mAb dose, neonatal Fc receptor (FcRn) expression and function, rates of pre-systemic catabolism, pre-systemic target engagement, and mAb formulation may contribute to IIV in mAb absorption following SC dosing. Numerous investigators have attempted to delineate the relative contribution of each of these determinants.

#### 2.1.1. Determinants of Convective Transport

mAb is administered to the SC site travel through the interstitium by convection and diffusion. From the interstitial space of the hypodermis, mAb may enter the systemic circulation via paracellular pores within capillaries, and/or via transit into and through the peripheral lymphatics, passing through regional lymph nodes, with subsequent entry into the systemic circulation via the central lymphatics (mainly via the thoracic duct). Lymph vessels are known to have an irregular basement membrane, where endothelial cells are devoid of tight junctions between the cells [3]. The paracellular clefts open in response to the hydrostatic pressure difference created from lymph flowing from terminal capillaries into larger vessels [4]. Due to absence of smooth muscles, lymphatic vessels have no vasomotor activity, but lymphatic capillaries have much larger diameters than blood capillaries. These structural characteristics of the lymphatics facilitate uptake of macromolecules up to a diameter of 100 nm [4], while the pore size of blood capillaries are much more modest (ranging 5–12 nm) [5]. In addition, the capillaries that collect lymph from the interstitial space are about ten times more distensible than blood capillaries [6]. Immunoglobulins (IgGs) are approximately 10 nm in length [7]; hence, absorption via lymphatics might be considered as the likely pathway for the uptake of mAbs from the SC space [8]. However, it is also proposed that endothelial cells of blood capillaries transport macromolecules via caveolar channels, the tubular-vesicular system, and/or additional transcytosis mechanisms, plausibly contributing to the SC absorption of mAb [9,10,11].

A review of literature has shown that there is a conflict in opinion regarding whether the dominant pathway of absorption of macromolecules from the SC space is via lymphatic transport or via the more direct entry into vascular capillaries. Starling, in 1896, was the first to suggest that a fluid rich in protein would have the same osmotic pressure as that of blood and due to restrictive capillary permeability, protein in interstitial fluid may be most likely to gain access to the vascular space via the lymphatics [8]. Early studies performed in dogs [12,13] supported Starling’s concepts and for many years the salient features of this theory were not disputed. It was later shown in humans that subcutaneously administered mAb localizes in the lymphatics [14,15]. In recent years, using model proteins, numerous studies have been conducted across different animal models, where the percentage of dose recovered in the lymph was estimated by cannulating the single efferent lymphatic duct exiting the lymph node proximal to the site of SC injection, or by non-invasive fluorescence imaging of the SC injection site and the corresponding draining lymph node. It is assumed that the selected efferent lymphatic duct is the only conduit for the draining lymph from the lymph node to the peripheral lymphatic system and that the selected lymph node is the sole node involved in transport from the injection site. Results observed from such work (Table 1) were not consistent between animal models and within animal models. Studies conducted in sheep, pig, and dog models agreed with the concepts proposed by Starling, as they showed that protein absorption from the SC site occurred primarily via the lymphatic system [16,17,18,19,20,21,22,23,24,25]. However, studies conducted in rabbits [26] and rats for a range of model proteins [27,28] showed minimal accumulation (<5%) of protein in lymph (collected via thoracic duct). These studies concluded that the lymphatic system did not contribute substantially to the absorption process. Contrarily to these findings, polyethylene glycol-protein conjugates and trastuzumab were found to be primarily transported to the lymphatics following SC dosing in rats [23,24,29,30]. In a series of studies conducted by Wu and co-workers in mouse models [31,32,33], using fluorescence based imaging, higher lymph node exposure of the protein was found following SC administration as compared to IV. However, in a subsequent investigation, the authors found that only 1% of infrared dye 800 CW labelled bevacizumab was taken up by the lymphatic absorption [34].

The lack of agreement in published reports could be due to dissimilarities in the lymphatic drainage pathways between animal models and due to differences in the selected site of injection within the same animal model. In studies conducted in sheep and dogs (Table 1), the dose was given in the inter-digital space/left hind leg, and lymph was collected via the thoracic duct (central lymph) or via the efferent duct of the popliteal lymph node (peripheral lymph). This approach is commonly employed in these animal models, as cannulation can be conveniently performed very closely to the point where lymph predominantly enters the venous circulation. In rat studies [27,28], where minimal recovery of administered protein was found in the lymph, the macromolecule was administered at lateral side of thigh, and the cannulated lymphatic vessel was the thoracic duct. Mappings of rat lymphatic drainage pathways by Tilney et al. [35] have shown that lymph drains from the thigh region to branchial, inguinal, and axillary nodes into the subclavian duct, bypassing the thoracic duct. In the rat studies [23,24,29,30] where lymphatic absorption was found to contribute to SC absorption, the site of injection was in the lower hind leg, from where the lymph is found to drain to the thoracic duct [35]. In the surveyed mouse studies [31,33,34], mice were injected in the front footpad and the drainage from the axillary lymph node was monitored. Moreover, in the studies [26,27,28] that concluded that the lymphatic system had minimal contribution to protein absorption following SC injection, central lymph was collected via the thoracic duct and loss of protein during lymph transport was not considered.

The cumulative absorption of a macromolecule given SC via lymph is found to increase with the molecular weight (MW) of the protein (strong linear correlation, *r*^2^ = 0.998) [16,17]. Proteins of MW greater than 16 kDa were found to be absorbed primarily via the lymphatics with >50% recovery in the lymph after SC administration. Dedicated studies conducted in rat [36] and mouse models [31,37] have consistently given similar linear relationships, suggesting that MW is an important determinant of lymphatic uptake. But MW has not been found to affect the overall bioavailability of the protein [38].

In humans, variability in the lymph flow rate has been reported and found to be related to diurnal rhythm, exercise, limb movement, hyperthermia, massage, and pressure [39,40,41,42]. Severe weakening of the lymphatic pump has been observed with aging, leading to the diminishment of the lymphatic contraction amplitude and frequency [43]. For mAbs like canakinumab [44,45], denosumab [46,47] and anti-interleukin receptor IL-4Rα (AMG 317) [48], age was found to be a negative covariate for the absorption rate constant. A decrease in lymph flow rate with age is likely to explain the decrease in rate of absorption. In inflammatory disease conditions, elevation of cytokines may significantly alter lymph flow and decrease the integrity of the lymphatic barrier [49]. Cytokines like interleukin-6 (IL-6), tumor necrosis factor-α (TNF-α), and interleukin-1 beta (IL-1 β) have been found to decrease systemic lymphatic propulsion [50], probably due to the dilation of vessels in response to inflammatory cytokines and stagnant pooling of the lymph. The significance of these cytokine-induced changes on the SC absorption of therapeutic proteins has been not been evaluated.

In humans, the time to peak in SC absorption often ranges from 3 to 8 days. Experimental evaluations have found sustained retention of protein at the SC site of injection (e.g., the half-life of protein loss from the SC injection site to be 33.4 h for 131Iodine labeled albumin in humans [51] and 6.81 h for fluorescence labelled bevacizumab in mice [34,52]). The lymph flow rate is reported to be in the range of 3 mL/min in the largest thoracic duct, and 0.15–0.6 mL/h in superficial leg lymphatics in humans [6]. Following intradermal injection of 99mTc-human IgG in humans, the lymph transit time from the hand to axilla was reported to be 9.6 ± 7.2 min [53]. Although the lymph flow rate is slow, and lymph transit time is long, lymph transit time is much faster than the calculated residence time for SC absorption. As such, lymph flow rate is unlikely to be the rate limiting step in protein absorption from the SC site; rather, the interaction of protein with extracellular matrix (ECM) and slow interstitial transport are most likely responsible for the observations of slow SC absorption [52].

The thickness of the hypodermis decreases with age [54] and increases with body weight [55]. The composition of the ECM in adipose tissue is altered in obesity and in disease conditions including diabetes and dermal dysfunction [56]. Increased adipose tissue has been associated with poor lymphatic drainage. Both in preclinical [23] and clinical studies, body weight has been found to be a negative covariate with bioavailability for therapeutic proteins such as human growth hormone [57] and erythropoietin [58]. For SC dosing of human growth hormone, obese women were found to have lower bioavailability, lower maximum serum concentrations (Cmax), and lower area under the plasma concentration v. time curve (AUC) [57].

#### 2.1.2. Role of Anatomical Site for Subcutaneous Injection

The site selected for injection has been found to influence the rate and extent of SC absorption for numerous therapeutic proteins in clinical use, including insulin [59,60], human growth hormone [61] and erythropoietin [62,63]. For example, in the clinic, the rate of SC absorption of insulin was slower (longer half-life of absorption) following deltoid and femoral administration in comparison to abdominal administration. Slower rates of absorption may lead to greater degradation of insulin at the injection site, resulting in a lower Cmax [59,60]. In preclinical studies conducted in rodent and sheep models [64,65,66,67], SC injection at inter-digital sites, the foot, and foot pads showed higher macromolecular SC absorption/bioavailability when compared to SC injection at the lower back, the shoulder, and the abdominal region. In humans, the depth and nature of the SC space is known to vary with anatomical location, race, age, gender, body mass index (fat composition), pigmentation, and smoking habits [68,69]. However, in dedicated clinical studies carried out for mAbs like golimumab and sirukumab, differences in the injection site (upper arm, abdomen and thigh) and race (between Japanese and Caucasian) were not found to influence SC PK significantly [70,71,72].

#### 2.1.3. Role of Neonatal Fc Receptor

In SC absorption, FcRn has been suggested to have two main contributions: protection of mAb from catabolism and participation in transcytosis of mAb across the vascular endothelium. FcRn is considered to be a primary determinant of SC bioavailability of IgG/mAb. Garg and Balthasar [73] showed a significant decrease in mean SC bioavailability of IgG in FcRn knockout mice compared to control mice (82.5 ± 15.6% vs. 28.3 ± 6.9%, *p* < 0.0001). A trend towards saturation of capacity limited FcRn-mediated protection was observed with an increase in dose, where bioavailability decreased with increasing dose in wild-type mice (expressing functional FcRn). Deng and co-workers [74,75] strategically engineered a range of mAbs with different binding affinities to FcRn at pH 6. They found that mAb with higher FcRn binding affinity at pH 6 (with no binding at pH 7.4) had higher bioavailability in mice than the corresponding wild-type or in comparison to a variant with lower binding affinity (at pH 6). However, Datta-Mannan et al. [76] failed to see a benefit in bioavailability of similarly engineered mAb variants in monkeys, despite improvements in clearance and half-life. Using mathematical modeling, Kagan and co-workers [66,67] attempted to delineate the relative contribution of FcRn-mediated protection from catabolism and FcRn-mediated transcytosis and showed that FcRn-mediated transcytosis is the main determinant of SC bioavailability. There is no experimental evidence favoring the contribution of one FcRn transport pathway over the other; however, the available data clearly demonstrate that FcRn transport is a main determinant of mAb SC absorption.

#### 2.1.4. Role of Pre-Systemic Catabolism

Pre-systemic catabolism at the SC site and during lymphatic transport has been reported for therapeutic proteins like insulin [77,78,79], human growth hormone [20], and erythropoietin [23]. The act of injection induces a transient injury that incites the release of proteases and peptidases from resident fibroblasts into the SC space, which is typically devoid of such repertoire of enzymatic activities [69]. Proteases may also enter SC site via blood capillaries. Concentrations of proteases including metalloproteinase, calpain, cathepsin, and caspase are elevated in pathological conditions like cancer, inflammation, diabetes, obesity, osteoporosis and hypertension [80]; the significance of these changes on the SC bioavailability of therapeutic proteins has not yet been evaluated.

Enzymatic catabolism may be responsible for the observed incomplete SC bioavailability of mAb. Wang et al. [23] analyzed SC tissue and lymph node homogenates, finding catabolites of PEG linked erythropoietin at the SC site and at the lymph node, suggesting catabolism of the protein during lymphatic transport. The fragments detected at early time points in the thoracic lymph were suggested to result from catabolism during lymphatic transport. Previously, Charman et al. [20] had also found evidence of catabolism for human growth hormone during lymphatic transport. A similar experimental evaluation for mAbs has not been yet conducted; however, it is likely that the incomplete bioavailability observed with slower rate of absorption may be due to catabolism that may occur at the injection site or/and during lymphatic transport.

#### 2.1.5. Role of Target at Subcutaneous Site

Antibody interaction with the target at the SC site may mediate mAb elimination (i.e., pre-systemic target-mediated elimination). In such cases, the application of standard non-compartmental methods may be expected to yield inaccurate estimates of bioavailability (due to the inherent nonlinearities of target-mediated elimination). On the other hand, the influence of target-mediated pre-systemic elimination may be of minor importance if the administered dose far exceeds the binding capacity of target. For example, assessments of anti-CD4 mAb in human CD4 transgenic mice showed that the presence of target CD4 at the SC site led to a dose dependence in SC bioavailability with no systemic absorption at low dose (0.4 mg/kg) and with high systemic bioavailability following a high dose (100 mg/kg) [81]. Similar results have been shown for rituximab (RTX), which binds to CD20 on lymphocytes [82].

#### 2.1.6. Role of Antibody Dose

Using wild-type rat and mouse models (i.e., devoid of human CD20), Kagan and co-workers [65,66,67] showed that SC bioavailability of RTX was inversely correlated to the dose level, and the authors suggest that saturation of FcRn may lead to the observed decrease in bioavailability with increasing doses. They found that co-administration of non-specific IgG (500 mg/kg) decreased RTX exposure by 6.5-fold following 1 mg/kg SC RTX dosing, and by 2.6-fold following 10 mg/kg RTX SC [66]. Shah et al. also observed a similar trend of decreasing bioavailability with increasing doses [83]. Additionally, following a SC dose of 10 mg/kg, Kagan et al. estimated RTX bioavailability to be 44% (abdomen site) and 31.2% (back site) in the rat model [65], and 77% (abdomen site) and 88% (back site) in the mouse model [67]. Shah et al. employed a modeling approach to estimate the bioavailability of a model murine IgG1 mAb, 8C2, to be 99.7% at 10 mg/kg and 48.6% at 3.77 g/kg [83]. Although both groups speculated that the observed dose dependency was due to saturation of FcRn, which protects IgG from intracellular catabolism, the work of Kagan and co-workers [66] suggested that saturation of FcRn occurred at lower doses than suggested by the analyses of Shah et al. The differences between the analyses may be related to differences in model assumptions and/or related to differences between murine FcRn interaction with 8C2 (murine IgG1) and RTX (chimeric IgG1).

#### 2.1.7. Role of PEGylation and Co-Formulation Strategies

PEGylation of therapeutic proteins has been considered as a strategy to improve SC absorption and decrease pre-systemic catabolism. Studies have shown that PEGylation improves lymphatic uptake of smaller proteins like polylysine dendrimers [29], erythropoietin [23] and interferon α [84] as PEGylation augments the MW of the protein, increases lymphatic uptake and improves bioavailability. For larger proteins like mAbs, PEGylation of trastuzumab with a single linear 40 kDa methoxy PEG was found to increase the bioavailability from 86.1% to 100% [85]. Although the bioavailability improved, compared to IV, the mono-PEGylated trastuzumab had an accelerated plasma clearance following SC absorption, possibly due to the formation of immunogenic response against the formulated moiety. PEGylation was also found to restrict basolateral to apical vascular transport, decrease binding to HER2, and, possibly interfere with FcRn binding [85].

Co-formulation of mAb with hyaluronidases like the Food and Drug Administration (FDA) approved recombinant human PH20 enzyme (rHuPH20, Hylenex^®^ recombinant) has shown clinical benefit in improving the SC absorption of trastuzumab and RTX [66,86,87,88], with a low incidence of immunogenicity (3–18%) [89]. rHUPH20 cleaves hyaluronan, disrupts the channels of the ECM, decreases the viscosity of the gel-like matrix, reduces interstitial pressure, and thus allows administration of volumes greater than 1–2 mL [86]. Apart from increasing the volume of injection, Kagan et al. showed that hyaluronidase increases rate of absorption and bioavailability of RTX [66], possibly by decreasing resistance from the ECM. Other formulation excipients like albumin (to manipulate oncotic pressure) [26] and hypertonic buffer [90] have been used to alter and enhance the bulk movement of fluid in the interstitial space and facilitate the absorption of therapeutic proteins via lymphatic uptake.

### 2.2. Mechanisms of Antibody Distribution

Following IV administration, mAb in plasma may extravasate via convection, diffusion, and pinocytosis. Extravasation from blood capillaries by paracellular transport is likely to be the primary mechanism of transit from blood to interstitial fluid for most proteins. Capillaries found in connective tissue, skin, muscle, fat, nervous tissue, and brain are mostly continuous with tight junctions between vascular endothelial cells. Fenestrated capillaries (renal glomeruli, intestinal villi, endocrine glands) and sinusoids (found in liver, spleen, bone marrow) have intercellular clefts of 30–80 nm and 100 nm respectively [91]. Due to their size (10 nm in length), polarity, and lipophobicity, mAbs are considered to be mainly dependent on convection and to a lesser extent, on diffusion for transvascular and transcapillary transport. Consistently with Poiseuille’s equation for hydrodynamic flow, convective transport is thought to be dependent on hydrostatic and osmotic pressure gradients between vascular and interstitial fluids [92].

After extravasation, mAb may distribute through interstitial fluid via diffusion and convection. Cells within tissues may internalize mAb within interstitial fluid by receptor-mediated endocytosis (e.g., mediated by Fc gamma receptors, membrane bound target antigen, etc.) or by fluid-phase endocytosis. In the absence of efficient receptor-mediated endocytosis (e.g., for mAb exhibiting target-mediated disposition), fluid phase endocytosis, which may be considered to be a non-specific mechanism, likely serves as the primary pathway of cellular entry of most mAb within most tissues. Following endocytosis, mAb within cellular endosomes is exposed to acidic pH that favors mAb binding to endosomal FcRn. Binding to FcRn protects mAb from lysosomal degradation and facilitates exocytosis to the interstitial space. mAb may be removed from the tissue interstitium by the drainage of interstitial fluid into lymphatic capillaries, which then eventually drain into the venous circulation. Due to differences in the porosity of blood and lymph capillaries, there is a greater restriction to mAb uptake into interstitial fluid when compared to mAb uptake into lymph fluid. As such, the convective elimination clearance is typically greater than the convective uptake clearance of mAb in tissue, and, consequently, mAb concentrations in tissue interstitial fluid are typically much lower than mAb concentrations in plasma.

Due to the role of FcRn in limiting the intracellular catabolism of mAb, the transporter contributes substantially to the long biological half-life of mAb. FcRn is composed of a transmembrane α-chain (heavy chain) and β2-microglobulin (14 kDa, light chain), closely resembling the structure of major histocompatibility complex class I molecules [93]. Investigations conducted with FcRn knockout mice [94], with evaluation of skin and muscle tissue samples, showed that IgG mAb co-localize with vascular endothelial cells, whereas IgG mAb demonstrated a more extensive and more homogenous distribution throughout interstitial fluid in wild-type mice (with functional FcRn). For highly perfused organs like the heart, lung, liver, spleen, GI tract and kidney, tissue to plasma exposure ratios of mAb were found to be similar in both wild-type and FcRn knockout mice. Hence, FcRn does not appear to be a critical determinant of the tissue selectivity of mAb distribution [94]. Similar results were obtained by Chen et al. in FcRn α-chain knockout mice [95]; however, higher tissue to plasma ratios were reported for the liver, spleen, and kidney of FcRn knockout mice vs. wild-type mice.

#### Tissue-Specific Properties Affecting mAb Distribution

Target: For many mAb, mAb-target binding influences, the rate and extent of mAb tissue distribution. For such mAb, tissue distribution is a function of the expression of target in tissues, the affinity of mAb-target binding, the fate of mAb-target complexes, and the accessibility of mAb to target (i.e., tissue blood flow, tissue vascular porosity, etc.).

Vascular porosity: The nature of paracellular pores in vascular capillaries is associated with substantial tissue-to-tissue heterogeneity. Antibody distribution in the brain is quite limited. Several preclinical studies have indicated that brain: plasma concentration ratios of monoclonal antibodies are 1:500, which is far below the values found for other tissues. Although several mechanisms may contribute to the low exposure of mAb in the brain, the tight junctions of the blood brain barrier, which limit the transcellular movement of macromolecules, are thought to play a major role [96]. Other mechanisms that may contribute are the rapid turnover of interstitial fluid within the brain, which enhances the convective elimination clearance of antibody [97], and the possible role of receptors (e.g., FcRn) in facilitating the efflux of antibody from brain fluids, and potentially from other tissues (discussed below) [98].

Barriers associated with solid tumors: Due to disorganized cell growth, solid tumors present unique barriers to mAb distribution. Tumors often lack functional lymphatics, with abnormal blood vessels that are highly irregular, with increased porosity, complex branching patterns, and with poorly vascularized regions (especially in large solid tumors). The blood flow rate is sluggish and unstable leading to nutrient and oxygen deprivation, and resulting in areas that are acidic and necrotic [99]. The ECM in tumors has been found to retard the movement of solutes. Additionally, due to the lack of functional lymphatic vessels and inefficient drainage of interstitial fluid, solid tumors often exhibit high interstitial fluid pressure, which minimizes the hydrostatic pressure driver for mAb extravasation in tumors by convection [100]. On the other hand, tumor blood vessels are reported to exhibit a ten-fold wider diameter of paracellular pores compared to normal vessels, which decreases sieving and, thus, enhances the efficiency of paracellular transport.

### 2.3. Mechanisms of Antibody Elimination

Primary putative mechanisms of mAb elimination include: (a) intracellular catabolism following fluid phase pinocytosis, (b) intracellular catabolism following target- or receptor-mediated endocytosis, (c) intracellular catabolism following cellular uptake of immune complexes (i.e., following interaction with host ADA), and, to a very limited degree, (d) excretion into the bile and urine. As indicated above, intracellular catabolism of IgG mAb is modulated by the function of the FcRn receptor. The role of FcRn in mAb disposition has been evaluated and characterized by the use of high IgG doses to saturate FcRn, engineered mAb with increased and decreased FcRn affinity, anti-FcRn antibodies and peptides, and through the use of FcRn-deficient mouse models. Hansen and Balthasar [101] showed that high-dose intravenous immunoglobulin (IVIG) therapy, which is utilized clinically for the treatment of autoimmunity, leads to a dose-dependent increase in the clearance of a model monoclonal antibody, 7E3 (an antiplatelet IgG1 mAb), with a 2-fold increase in clearance following 2 g/kg IVIG. Specific anti-FcRn inhibitors were shown to be more efficient in achieving increased antibody elimination; for example, 60 mg/kg dosing of the anti-rat FcRn mAb 4C9 led to a doubling in the clearance a model IgG antibody in rats [102]. Several groups showed a 10–15-fold increase in IgG clearance in FcRn-deficient mouse models [103,104,105]. mAbs engineered for decreased FcRn binding affinity show more rapid elimination [106], and several reports have shown that mAb clearance may be decreased through engineering mAb for increased FcRn affinity [107,108]. These studies have strongly supported a key role for FcRn as a determinant of IgG elimination kinetics.

Due to the large molecular size of IgG, glomerular filtration and biliary excretion appear to play a minor role in mAb clearance. However, FcRn is expressed within the kidney, and it may play a role in IgG reabsorption, as suggested by Haymann et al. [109]. Additionally, FcRn expressed on podocytes has been suggested to be involved in increasing the renal clearance of filtered IgG, FcRn expressed on the endothelium of the renal blood vessel has been proposed to transport IgG into the kidney interstitium, and FcRn expressed on the brush border of proximal tubular epithelial cells may modulate IgG elimination in the urine [110,111].

In addition to the role played by target interactions in mAb distribution (discussed above), target binding often mediates mAb elimination. Key determinants of target-mediated mAb clearance include mAb-target affinity, the extent of target expression, kinetics of target turnover, the fate of the mAb-target complex, and the accessibility of target to mAb (e.g., in plasma or in tissue fluids) [97].

Interaction with Fc gamma Receptors (FcγRs) may initiate endocytosis and catabolism of monomeric IgG mAb or mAb immune complexes. The significance of FcγRs in mAb clearance is expected to be modulated by the extent of saturation of FcγRs by monomeric endogenous IgG, as IgG concentration in plasma (typically 65 µM) [97] is far above the equilibrium dissociation constants between IgG and FcγRs (10 nM–1 µM) (Table 2). Preclinical studies with selected mAbs suggest that FcγRs may play a minor role in mAb clearance [112]; however, the influence of gamma receptors would be expected to be increased in conditions of depleted concentrations of endogenous IgG, the development of immune complexes (e.g., between mAb and ADA), and in conditions when mAb has been engineered for high affinity binding to gamma receptors [113].

ADA that bind therapeutic mAb have been shown to dramatically increase mAb clearance [113]. mAb-ADA immune complexes may be rapidly internalized by cells of the reticuloendothelial system through FcγRs-mediated endocytosis (discussed above). Additionally, it has been suggested that the red blood cells may bind immune complexes, and then deliver the complexes to Kupffer cells in the liver, which then ingest and catabolize the complexes [114,115,116].

## 3. Variability in Determinants of Antibody Disposition

### 3.1. FcRn Gene Polymorphism and Expression

FcRn has an undeniably crucial role in regulating IgG clearance and homeostasis. Genetic mutation and polymorphism can affect the expression and function of FcRn and, consequently, determine the degree of exposure of therapeutic antibodies. Preclinical studies have evaluated the disruption of the FcRn transmembrane α-chain (heavy chain) and β2-microglobulin (14 kDa, light chain) [93]. Disruption of the β2-microglobulin gene has resulted in unusually short half-lives of IgG [104,121] and decreased protection from catabolism [105,122] in β2-microglobulin deficient mice. Similar results were obtained for the FcRn α-chain knockout mice as well [95]. In humans, individuals with mutations within the β2-m gene sequence are reported to have familial hypercatabolic hypoproteinemia with severely reduced serum concentrations of IgG and albumin [123].

The human FCGRT gene (14 kb) encodes the heavy chain of FcRn and it is located on chromosome 19 [124]. FCGRT gene polymorphism has been shown to result in effects on FcRn expression, changes in FcRn function, and alteration of FcRn binding capacity in several animal species. Haplotypes identified in the bovine FCGRT locus were found to be significantly correlated with the concentration of serum IgG observed in neonatal calves [125]. Polymorphism was also found to influence the IgG content found in bovine colostrum [126] and haplotypes identified in the β2-m gene were associated with variability in IgG concentrations in newborn calves [127]. Similarly, polymorphism in porcine FcRn gene was found to be associated with variability in serum antibody concentrations [128]. In sheep, IIV in colostrum IgG concentration was attributed to the genetic polymorphism in the Fcgrt gene [129]. In humans, five alleles were identified in the variable number of tandem repeats (VNTR1-VNTR5, 37-bp-long motif) region within the FcRn promoter [124]. VNTR3/VNTR3 is the most common genotype. Monocytes from individuals homozygous for VNTR3 displayed increased binding to human IgG, suggesting that polymorphism can influence the transcription of the α-chain causing differences in IgG-binding capacity [130]. Passot and co-workers reported that patients homozygous to VNTR3 has lower distribution clearance of cetuximab than patients with VNTR2/VNTR3 and VNTR3/VNTR4 phenotype (*p* = 0.021) [124]. Similarly, Billiet et al. reported that inflammatory bowel disease patients with VNTR2/VNTR3 genotype were found to have 14% lower infliximab AUC and 41% lower adalimumab AUC concentration compared to patients homozygous for VNTR3/VNTR3 (*p* = 0.03) [131]. Recently, Caulet et al. reported that volume of distribution of bevacizumab was significantly higher in VNTR3/VNTR3 patients (*p* = 0.039), as compared to other genotypes [132].

### 3.2. Fc Gamma Receptor Expression and Polymorphism

FcγRs are expressed on a variety of effector cells including mast cells, natural killer (NK) cells, macrophages, neutrophils, basophils, dendritic cells, monocytes, platelets and are crucial to effector machinery. IgG engagement of Fc receptors FcγRI, FcγRII (a,b,c) and FcγRIII (a,b) initiates inflammatory responses, resulting in the activation of platelets and mast cells, degranulation of neutrophils, antibody dependent cell cytotoxicity (ADCC), and phagocytosis of targets [133]. IgG-FcγR interaction also facilitates the release of pro-inflammatory mediators like histamine, eicosanoids, cytokines, and chemokines [133]. FcγRs play a critical role in the functioning of the humoral immune system, mediating inflammatory response, and clearing of immune complexes [134,135]. The FcγRs vary in terms of their expression on cell types, binding affinity to IgG subtypes, and activation by immune complexes (Table 2). FcγRI (CD64) is a high-affinity receptor that binds to monomeric IgG and is critical to T cell mediated immunity [133]. FcγRII (CD32) and FcγRIII (CD16) are low affinity receptors that bind with high avidity to multimeric immune complexes [136]. FcγRIIa has an immunoreceptor tyrosine-based activation motif (ITAM), while FcγRIIb has an immunoreceptor tyrosine-based inhibitory motif (ITIM) that exerts inhibitory function via the ITAM pathway [133]. IgG1-FcγRIII receptor interaction occurs via CH2 and CH3 domains results in ADCC [137], while interaction between IgG1 and C1q of the complement system occurs via the CH2 domain activates the complement cascade.

Heterogeneity in FcγRIIa, FcγRIIIa and FcγRIIIb has been reported and the corresponding polymorphisms—FcγRIIA-R/H131, FcγRIIIA-V/F158 (also called FcγRIIIA-V/F176) and FcγRIIIB-NA1/NA2—are found to alter binding to IgG and consequently affect IgG effector functions [134]. The H131 allele has higher binding for IgG2 immune complexes compared to R131, and heterozygotes tend to have intermediate function. About 50% of healthy European and African subjects have the R131 allele and the frequency goes down to 30% in individuals of Asian ancestry. Among the allelic variants of FcγRIIIA, the V allotype has higher avidity for IgG1 and IgG3 while the F allotype is represented in higher frequency healthy individuals of Asian (68%) and European/African ancestry (58%). Individuals homozygous for the NA1 allele are found to have neutrophils with more robust capacity for phagocytosis compared to individuals with NA2 allele. NA2 has a frequency of 65% in European and African individuals. FcγR polymorphism has been found to have differential effects on an individual’s susceptibility to various inflammatory and pathologic diseases [138]. The exact role of FcγRs in disease manifestation is unclear but it has been suggested that harboring a certain FcγR phenotype could be risk factor in development of specific diseases [139]. For example, the FcγRIIa-H131 genotype was found to be associated with an increased production of the pro-inflammatory cytokine IL-1β by mononuclear cells, leading to inter-individual differences in the risk for acquiring periodontitis [140]. In multiple studies carried out in diverse ethnic populations, significant associations were found between FcγR polymorphisms and inter-individual differences in susceptibility, prevalence, and prognosis of diseases like systemic lupus erythematosus (SLE) [141,142,143,144], rheumatoid arthritis (RA) [145,146], immune thrombocytopenia [147], Guillain–Barré syndrome [148,149,150], myasthenia gravis [151] pediatric autoimmune neutropenia [152], IgA nephropathy [153], inflammatory myopathies [154], anti-glomerular basement membrane antibody disease [155], Kawasaki Disease [156], periodontitis [157,158], malaria [159,160], dengue [161], and sickle cell disease [162].

Prior studies done by Abuqayyas and Balthasar, using model mAb, have shown that FcγR expression has minimal influence on antibody plasma PK and tissue distribution [112,163]. Alteration in FcγR binding also has not been found to affect mAb PK in cynomolgus monkeys compared to wild-type antibody [164]. FcγR polymorphism becomes a relevant clinical concern for mAbs like trastuzumab, cetuximab and RTX whose efficacy is dependent on the cytotoxicity resulting from mAb engagement of FcγRII and FcγRIII. Individuals with certain polymorphic FcγR phenotypes have effector cells with a higher affinity for IgG1 and ability to induce more potent ADCC at lower antibody concentrations; the allotypes do not seem to differ in intracellular signaling [165]. The polymorphic residues in FcγR co-localize with the docking sites of IgG Fcs and hence polymorphism modulates IgG binding interactions [166]. FcγRIIIa has a functional allelic dimorphism at amino acid position 158, resulting in three genotypes FcγRIIIa-158 V/V, FcγRIIIa-158 F/F and FcγRIIIa-158 V/F. Individuals harboring V/V genotype have a more effective ADCC via better binding of natural killer cells to Fc region of the mAb [167,168]. Populations with V/V phenotype patients were found to have better response to RTX in non-Hodgkin’s lymphoma (NHL) [165,169,170,171] with no influence on the clinical course of the disease or response to other chemotherapeutic drugs [172,173]. Similarly, FcγRIIA-R/H131polymorphism was found to predict response to RTX in NHL patients independent of FcγRIIIa polymorphism and patients with FcγRIIA-131 H/H showed better response [170,174]. Congy-Jolivet and co-workers confirmed that V/F158 polymorphism did not influence FcγRIII receptor expression in NK cells, rather the improvement in clinical outcome was associated with a higher affinity for IgG1 [175]. FcγRIIIA-V/F158 polymorphism was found to influence the clinical outcome of RTX therapy in Waldenström’s macroglobulinemia [176] and systemic autoimmune disease [177] but not chronic lymphocytic leukemia (CLL) [178]. The lack of influence of polymorphism in CLL has been suggested to be due to low contribution of ADCC in efficacy of RTX in CLL as well as low expression of CD20 in CLL [178]. Clinical evaluation of polymorphism require large cohort of patients with similar disease baseline and dosing regimen to accurately investigate associations between a genetic phenotype and therapeutic response. In most studies, the long-term implications of genetic polymorphism on mAb treatment is not evaluated and its effect on antibody PK is underreported or not carefully examined. Interestingly, in infliximab (IFX) therapy of Crohn’s disease, patients with V allotype have shown to be significantly better responders to mAb treatment [179,180]. In one of the first studies analyzing the effect of polymorphism on mAb PK, Ternant et al. showed that patients with V allotype had a higher elimination rate constant of 0.057 day-1 (versus 0.049 day-1 observed in F carriers, *p* = 0.0028). IFX treatment was found to be effective in V/V patients; however, the drug was cleared much faster, leading to underexposure and increased risk of relapse, especially in V/V subjects that had high disease activity [181]. Using population PK modeling, Ternant et al. also demonstrated that in CLL patients, the FcγRIIIa -158V/V genotype was identified as a significant covariate on the target-mediated elimination of RTX (*p* = 0.0016) [182]. This finding, however, contradicts an earlier report [178], which indicated that FcγRIIIa polymorphism did not impact RTX effects in CLL patients. For cetuximab, in vitro studies [183,184] suggested improved activity for the V/V genotype, while in vivo studies [185,186] showed that patients with the F/F genotype showed superior outcomes, indicating a possible involvement of other factors. However, FcγR polymorphism could possibly influence both mAb pharmacodynamics (PD) and PK.

### 3.3. Target Properties

Antibody therapeutics have opened new possibilities for a repertoire of prospective targets. mAbs are currently employed for four broad applications: neutralizing toxins, mediating cell destruction, altering cell function and facilitating drug delivery thus covering a diverse array of targets like venoms, toxins, endogenous ligands, drugs, cell surface receptors, trans-membrane receptors, extracellular proteins, substrates and metabolites [113,187]. Advances in antibody-drug conjugation strategies may further expand the utility of antibody therapeutics to untapped intracellular targets [188]. Target proteins may be broadly classified as soluble antigens and as membrane-bound antigens. Antibodies against soluble antigens are typically, but not always, found to exhibit linear, dose-proportional PK, while antibodies that bind to cell-associated antigens often exhibit non-linear, dose-dependent PK [189]. The influence of drug binding to target on drug PK (i.e., target mediated drug disposition (TMDD)) is well appreciated through the literature, and the PK/PD implications of TMDD are now considered throughout the process of mAb development. Modeling and simulation strategies have been used to investigate mAb-target interaction, to understand the pharmacology of the system, and gain insight into the target properties controlling antibody disposition [190]. The TMDD model introduced by Mager and Jusko [191] and its numerous variations with quasi equilibrium, quasi steady-state, and Michealis Menten approximations [192,193] have been widely used in population modeling approaches for mAb therapeutics.

In one of first published investigations of the effects of target expression on mAb PK, Lammerts van Bueren et al. reported that higher plasma of concentrations of an anti-epidermal growth factor receptor (EGFR) antibody (2F8) were required to saturate EGFR in animals bearing tumors with high expression of the target. The target was shown to serve as an ‘antigenic sink’ [194]. High target expression is desirable in many ways, as this often allows improved selectivity of antibody-based therapy; however, high antigen density also may impede antibody penetration (referred to as the ‘binding site barrier’), lead to rapid target-mediated elimination, and lead to a requirement for high mAb doses to achieve a desired degree of target occupancy [195]. Interestingly, the rates of target-mAb internalization and target turnover has been predicted to influence tumor penetration [196]. Rapid internalization and target-mediated clearance of mAb leads to an increased sink effect, further impeding antibody penetration and distribution within the tumor. Slower internalization rates may also facilitate ADCC and complement dependent cytotoxicity (CDC) mechanisms of mAb action, which proceed through the engagement mAb, via Fc domains, on the surface of tumor cells [197]. Target-specific variables like expression, internalization rate, turnover, shedding rate, and polymorphism, can govern the relationships of antibody disposition, efficacy, and dosing. Variability in the aforementioned target properties can be a source of inter-individual differences in PK and PD.

#### 3.3.1. Target Expression

Some targets are highly variable in their expression (Table 3). For readily accessible targets like soluble antigens, target concentration can been the basis for dose selection, while also serving as a marker for disease activity, efficacy, and prognosis. Takeuchi et al. found a ten-fold variability in baseline TNF-α concentration in RA patients (*n* = 327) ranging from 0.92 to 9.68 pg/mL. Patients with low baseline TNF-α concentrations responded to lower doses of IFX and they did not benefit with higher doses of IFX; alternatively, dose escalation was required for IFX efficacy in patients with higher baseline TNF-α concentrations [198]. The use of pretreatment measures of soluble target in plasma to facilitate dose selection may enable individualized therapy, allowing improved efficacy and safety. This type of strategy has been implemented for omalizumab (anti-IgE mAb) in asthma patients where doses are selected based on patient weight and baseline concentrations of IgE [199]. This approach helps to overcome the very significant IIV in baseline IgE concentrations, which were shown to range from 51 to 1692 ng/mL in a sample of 245 patients with severe persistent allergic asthma.

In an early clinical study done in NHL patients, mAb tumor uptake was found to be inversely proportional to tumor burden [200]. Koon et al. showed that differences in tumor burden could explain the differences in the clearance observed in CD25 positive leukemia patients (*n* = 10) treated with daclizumab (anti-CD25 antibody) [201]. In the past, tumor burden has been suggested as a useful metric to determine the extent of the disease, identify high risk patients, and predict prognosis [202]. Dayde et al. evaluated the effect of tumor burden on the concentration-response relationship of an anti-CD20 antibody, RTX, in syngeneic bioluminescent mice expressing CD20 [203]. Using PK/PD modeling, the authors demonstrated that high tumor burden led to low mAb exposure [203]. In a pre-clinical investigation performed by Boross et al., it was found that the mechanism of action of CD20 antibodies varied with tumor burden. A tumor with low antigen burden could be effectively be eliminated by CDC alone but a combination of effector mechanisms (ADCC, CDC, and apoptosis) was required for efficient removal of higher tumor burden [204]. Tumor burden was identified as a significant covariate in the phase I-II clinical trial of ofatumumab, an IgG1 anti-CD20 antibody that targets the membrane proximal epitope of CD20 in refractory CLL [205]. Similarly, inclusion of the baseline tumor size (5390 ± 19,100 mm^2^) partly explained the IIV observed in CLL and NHL patients (*n* = 678) treated with obinutuzumab, an anti-CD20 mAb having enhanced ADCC activity relative to RTX. Clearance of obinutuzumab was found to be affected by baseline tumor size [206]. Likewise, it was found that tumor burden increased the target-mediated clearance of trastuzumab in non-metastatic breast cancer patients [207]. Determination of baseline target concentration and tissue antigen burden in mAb clinical development could allow appropriate dose selection, improved development of mAb concentration-efficacy relationships, and improve our understanding of the inter-patient variability observed in therapeutic response.

#### 3.3.2. Target Shedding

Antigen shedding is a common feature of metastatic cancer cells [215]. In pre-clinical studies, shed antigen was found to alter antibody biodistribution and clearance, the antibody complexes with the circulating antigen, which is subsequently removed by liver and spleen, decreasing tumor uptake and efficacy [216,217,218]. It has also been highlighted that apart from circulating antigen, the antigen released within the tumor interstitium could be an undetectable barrier to antibody-based therapies [219]. In a preclinical study, Davies et al. showed that antibody tumor uptake was decreased by approximately 50% in the ovarian cancer xenograft mouse model having shed antigen in circulation compared to the corresponding xenograft that did not shed antigen [220]. In early studies carried out in ovarian cancer patients, there was supporting evidence of complex formation between shed antigen and therapeutic antibody [221]. McQuarrie et al. reported that if the antibody concentration was in excess of the shed antigen, mAb therapy was largely unaffected [222]. In a pre-clinical experiment, Pastuskovas et al. confirmed that if mAb concentrations markedly surpass the shed antigen concentration levels, mAb PK remained unperturbed [223].

#### 3.3.3. Target Turnover and Internalization

The turnover rate of cell membrane receptors depends on their biosynthesis and degradation rates, which may range from minutes to 100 h or more [224]. Receptor dimerization, activation, half-life, and degradation are regulated by the intrinsic properties of the receptor itself [225]. Turnover rates may be measured in vitro, for example, through pulse-chase experiments or SILAC (stable isotope labeling by amino acid in cell culture) [226]. Although the turnover rates for certain receptors like EGFR have varied substantially in literature reports [226,227,228], it is unclear whether this variability relates wholly or partially to the methods employed for quantification of receptor turnover, and there is some uncertainty regarding the extent of intersubject variability in receptor turnover, particularly with regard to healthy cells or subjects. In the context of disease, such as cancer and neurodegenerative disease, clear data are available that demonstrate substantial variation in receptor turnover. For example, the RET51 isoform of RET tyrosine kinase receptor, which is associated with increased oncogenic potential, has a three-fold higher turnover rate than the RET9 isoform [229]. The turnover rate of B cells, and B cell membrane proteins such as CD20, varies between 15.40% and 59.13% among NHL patients (as estimated by the proliferation index) (*n* = 72) [230]. In CLL patients, a significant reduction in B cell turnover rate has been observed in clinic compared to healthy subjects [231], and via histological assessments it has been shown CLL patients have significantly lower expression of CD20 as compared to NHL (approximately 6 fold difference, 14064 vs. 82726 molecules of soluble fluorochrome, *p* < 0.002) [208]. The median terminal half-life of RTX is lower in NHL patients (22 days, range 6.1 to 52 days, *n* = 298) as compared to CLL patients (32 days, range 14 to 62 days, *n* = 21) [232]; the reduced B cell turnover and decreased CD20 expression observed in CLL patients is most likely to explain these PK differences.

Relative to the turnover of the target protein in the absence of mAb, the internalization rate of antibody-target complex is often faster [224]. In the case of some mAbs like trastuzumab, the increased rate of internalization of the receptor in complex with mAb leads to the downregulation of the receptor [233]. Differences in receptor turnover rates and internalization rates between subjects is difficult to assess experimentally; however, in some cases, modeling approaches may allow a priori predictions. Using Michealis Menten and TMDD models, Krippendorff et al. showed that the extent of non-linearity in antibody disposition is dependent on receptor density and the rates of mAb-receptor internalization. Non-linearity is higher for systems with higher receptor density and faster internalization [234].

#### 3.3.4. Target Heterogeneity

Substantial heterogeneity in the expression of tumor-specific antigen has been observed in cancer patients, causing major obstacles in diagnosis and treatment [235,236]. Antigenic heterogeneity resulting from phenotypic instability and clonal destabilization is suggested to be integral to cancer pathology to circumvent immune surveillance [235,236]. Clonal heterogeneity is found to follow the Darwinian model of evolution, leading to clinical resistance to targeted therapies [237]. Considerable antigenic variability has been observed in tumors obtained from patients having breast [238,239], lung [235], ovarian [239,240,241], prostrate [242], and head and neck cancer [243]. Apart from inter-tumoral heterogeneity, intra-tumor heterogeneity has also been reported in many human tumors [243,244,245,246,247,248,249].

Cetuximab and panitumumab are anti-EGFR mAbs effective in subset of colorectal cancer patients with wild-type KRAS. In clinical investigations, 38–60% patients initially harboring wild-type KRAS were found to acquire secondary resistance to EGFR blockade, 5–6 months post-treatment initiation, by developing mutant KRAS [250,251]. It is possible that the mutation resulted from de novo acquisition or expansion of a pre-existing resistant sub-clone. Montagut et al. identified a missense point mutation arising in the EGFR ectodomain during cetuximab treatment preventing mAb binding and leading to resistance; the mutation did not affect panitumumab binding [252]. Given the mechanisms involved, it is likely that the development of resistance to EGFR targeted therapy is a convergent phenotype, i.e., cancer cells adopt multiple mechanisms to resist the given drug.

In the case of trastuzumab, less than 30% of HER2 positive patients are responsive, and the remaining fraction exhibit ab initio resistance to mAb monotherapy [253]. Unfortunately, 74% of patients of the initial responders acquire secondary resistance during the course of the 5–9-month treatment period [254]. Multiple mechanisms of resistance have been proposed for trastuzumab, including co-expression of mucin1/mucin4 that hinders mAb binding [255,256] and activation of alternate signaling pathways via compensatory receptors such as EGFR [257], HER3 [258], insulin like growth factor receptor [259] and overexpression of MET receptor tyrosine kinase [260].

#### 3.3.5. Target Polymorphism

Target polymorphism has been explored as a source of inter-individual variability. Some individuals respond rapidly to a course of therapy while others remain partially responsive or completely non-responsive. Genetic polymorphism may predispose an individual to a certain treatment outcome. Differences observed in mAb clinical efficacy in patient populations are not clearly understood, especially in chronic inflammatory diseases. For example, in luminal and fistulizing Crohn’s disease patient populations, an average of 19.5–31.6% are partial responders and 25.6–28.7% are non-responders to IFX therapy [261]. Hlavaty et al. investigated the influence of FasL/Fas system and caspase-9 polymorphism on patient response, and found that Fas ligand −843 TT genotype was strongly associated with lack of response to IFX therapy [262].

Attempts to assess target polymorphism has been pursued as a strategy to predict patients that are likely to respond to mAb treatment. SNPs in gene promoter regions, introns, and gene sequence have been found to be associated with variability in cytokine synthesis [263]. Certain TNF-α and IL-10 haplotypes were associated with a higher production of TNF-α and IL-10 in Crohn’s disease and ulcerative colitis [264], possibly explaining the differences observed in baseline target concentrations and response observed in patients. Medrano et al. found an association between patient response to IFX and polymorphism in TNF receptor superfamily (TNFRSF1B) in Japanese Crohn’s disease patients [265]. Similarly, in RA, polymorphism found in IL-6-R receptors rs12083537, rs2228145, and rs4329505 were found to predict of response to tocilizumab therapy [266]. Many reports have found associations between polymorphic variants of cytokines and the efficacy of the corresponding mAb-based cytokine modulators, but to date, testing for polymorphism prior to therapy has not been practiced in the clinic.

### 3.4. Anti-Drug Antibodies

Although the determinants of immunogenicity of therapeutic mAb are not well understood, humanization of the primary sequence has enable reductions in immunogenic risk [267]. Product and process related impurities (degradation, oxidation, contaminants, conformational alterations, aggregates, micelles, excipients) contribute to immunogenic potential [268], but other factors (glycosylation/pegylation, route of administration, dosing interval, and genetic/disease/immune status of the patient) may contribute to risk [269]. The direct consequence of immunogenicity is loss of efficacy and, in some cases, immune-related toxicity (anaphylaxis, cytokine release syndrome, infusion reactions, serum sickness) [270]. However, development of ADA is highly variable among patients and, in some cases, within patients (i.e., where ADA are produced transiently) [271]. For example, 25% of patients developed ADA against IFX in alkylosing spondylitis [272] and 33% in RA patients [273]. The efficacy was compromised in patients with low serum mAb; however, in the remaining patients, the drug was found to be effective. Similar observations were found for natalizumab, where 5–10% of Crohn’s disease patients developed ADA reducing efficacy and patients experienced infusion related reactions like uritcaria and pruritus [274]. The development of ADA is reported to impact mAb clearance and elimination half-life (Table 4). Ternant et al. quantified the influence of ADA on IFX PK and reported a 2.7-fold increase in clearance and a 34% decrease in elimination half-life in inflammatory bowel disease patients [275]. ADA is a clinically significant covariate for clearance of number of mAbs like golimumab [276,277], ustekinumab [278,279], anti-IL1β mab [280], daclizumab [281], amatuximab [282], atezolizumab [283] and benralizumab [284] (Table 4). For most of the above cited therapeutic mAbs, the patients developed ADA 12 weeks after initiation of therapy, in line with the known somatic hypermutation kinetics of IgGs [269]. Persistent ADA led to reduced efficacy due to reduced target binding, as well as clearance of immune complexes via the reticuloendothelial system and elimination by complement activation and Fc receptors.

ADA is more commonly observed in autoimmune diseases as compared to cancer, possibly due obvious differences in the nature of the diseases. The underlying mechanisms for immunogenicity and the sources of variability in patient immune response have not been clearly elucidated. Pre-clinical risk assessment carried out using transgenic animal models can, to a certain extent, inform assessments of relative immunogenicity, but has limited utility for the prediction of clinical immunogenicity [285]. Non-human primates have been shown to be poor predictors of human immune response [286]. Immune response varies from patient to patient and also within patients, as the concentration and nature (isotype, affinity) of the ADA distribution change with time and with continued drug dosing [269]. Mathematical models have shown utility in their ability to characterize ADA responses following different doses, and to develop relationships between drug exposure and the impact of ADA on drug clearance [287,288]. In clinic, increasing the mAb dose, co-administration of immunosupressants [289,290,291] and switching to a different mAb [292] are considered as strategies to overcome immunogenicity and have shown moderate benefit.

## 4. Common Covariates Identified in Population Pharmacokinetic Modeling

**Figure 1:**
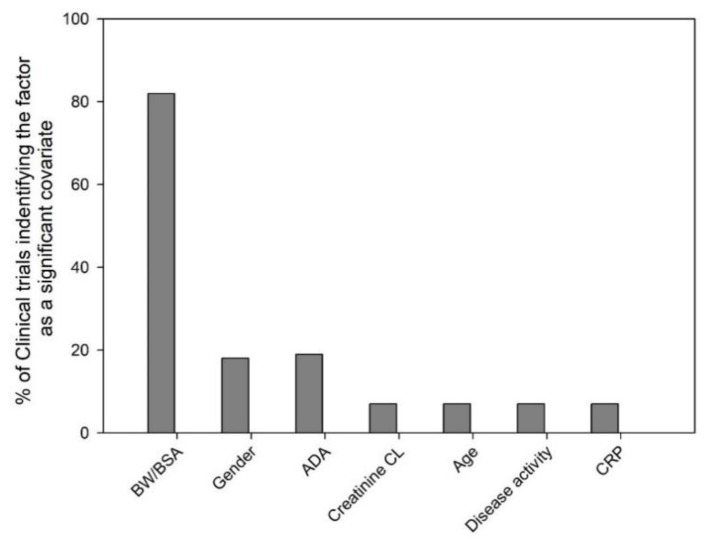
Evaluation of clinical trials for the most commonly identified significant covariates. The 100 clinical trials (phase I-III, years 2000–2018) that conducted population pharmacokinetic analysis were evaluated for the identified significant covariates. *Bars* represent the percentage of clinical trials that identified the variables- body weight/body surface area (BW/BSA, 82%), gender (18%), ADA (anti-drug antibody, 19%), creatinine clearance (CL, 7%), age (7%), disease activity (7%) and C-reactive protein (CRP, 7%) as a significant covariate.

We evaluated 100 mAb clinical trials (phase I-III, dose given I.V. bolus, I.V. infusion or S.C.) from 2000–2018 and the most commonly identified significant covariates on mAb PK have been depicted in Figure 1. Among the 100 clinical trials that conducted population PK modeling, the percentage of clinical trials that identified the variable as significant were: body weight/body surface area (82%), gender (18%), ADA (19%), creatinine clearance (CL, 7%), age (7%), disease activity (7%) and C-reactive protein (CRP, 7%). In this section, the biological relevance of several of the commonly considered covariates on mAb PK is discussed.

### 4.1. Body Size

Body weight (BW) and body surface area (BSA) are the most commonly identified covariates for mAb PK parameters via population modeling. Body size may affect non-specific clearance pathways and may be of particular importance for mAb with linear PK. BW/BSA based dosing has been applied as a strategy to limit exposure variability among subjects; however, superiority over fixed dosing has not been shown in several clinical evaluations. A review of 12 approved mAbs showed AUC variability of 42.4% for fixed dosing strategies and a mean variability of 44.2% for BW/BSA dosing strategies [300]. This finding may relate to the importance of target-mediated disposition to the PK of many mAb, and due to the lack of correlation of determinants of TMDD (e.g., receptor expression, receptor turnover) to body size [301]. However, it is important to note that exceptions have been reported. For example, investigation of panitumumab pharmacokinetics demonstrated that, among all examined covariates, BW had most influence on the non-linear clearance [302]. Fixed dosing strategies may be preferred for first-in-human trials; however, following the collection of data relating body size to PK parameters, there may be sufficient justification for BW/BSA based dosing for Phase II or Phase III clinical trials [300,303].

Body size and composition may impact mAb disposition by several biologically plausible mechanisms. Decreased lymph flow rates have been reported in obesity [304], which may influence the rate and extent of mAb distribution in tissues. Additionally, cancer patients with below-average body size may be more likely affected by cachexia [305], which may increase global rates of protein endocytosis and catabolism (increasing mAb clearance). Of course, body size is expected to correlate to physical volumes (e.g., plasma volume, interstitial fluid volume) that are determinants of mAb distribution.

Modeling strategies may aid in defining clinical dosing regimens that accommodate a broad spectrum of body size. Lebwohl et al. used a population modeling method to justify a fixed dosing strategy for ustekinumab in normal and obese psoriasis patients (45 mg for < 100 kg BW and 90 mg for > 100 kg BW) [306]. This regimen is employed clinically and comparable therapeutic outcomes have been reported for patients above and below the BW threshold [307]. Narwal et al. used a population PK model to support the use of a fixed dosing strategy for sifalimumab (anti-interferon alpha mAb) in SLE patients for a phase II clinical trial [308], and a similar approach was used to select and confirm the appropriateness of a fixed dosing strategy for pertuzumab (anti-HER2 mAb) [309]. Population PK/PD simulations have been used to suggest revisions to the omalizumab dosing protocol [310] and to support the extension of omalizumab dosing to patients who do not meet the criteria set in the approved omalizumab dosing table [311].

### 4.2. Sex

Sex is very frequently considered as a possible covariate in population PK modeling. Generally, females tend to have lower BW/BSA, higher body fat, lower muscle mass, lower glomerular filtration rates, and hormonal fluctuations during the menstrual cycle that may impact drug disposition. Other factors like use of hormonal contraceptives, and physiological changes associated with pregnancy and menopause, may contribute to sex-related biological differences [312].

Preclinical investigations indicate that Fc receptor expression and function may be modulated by a variety of hormones. Estradiol was found to significantly increase macrophage Fc receptor mediated clearance of IgG coated erythrocytes [313], and progesterone was found to reduce FcγR expression, activity, and macrophage dependent clearance [314,315]. Alteration in mammary FcRn expression during the course of lactation period has also been noted in a preclinical model, suggesting hormonal regulation of FcRn [316]. High doses of thyroxine was found to decrease FcRn expression in a dose- and time-dependent manner [317]. FcRn expression in conditions like hyperthyroidism have not been investigated but thyroid abnormalities can affect the number of individuals and is found to be 2–8 times more common is females than in males across age groups [318]. The available preclinical data suggest that gender-related hormones may influence FcRn and FcγR receptor expression and function, possibly leading to sex-based variability in mAb disposition and efficacy; however, clinical assessment is lacking.

In clinical studies, sex-based differences in PK parameters have been often attributed to differences in BW between male and female subjects. In general, males have higher BW and hence higher plasma volume than females, possibly explaining the relatively common finding that sex is a significant covariate on central volume of distribution (Vc) in PK models. For IFX, central volume was higher in males compared to females (2.3 L in male vs. 1.1 L in female) [275], and sex was identified as a significant covariate on Vc in inflammatory bowel disease, ankylosing spondylitis [319], and in ulcerative colitis [320]. In another clinical trial for IFX, sex was significant covariate on both Vc and clearance, where clearance was 35% higher in males compared to females [321]. Similarly, tocilizumab clearance in women was 16% lower compared to men [322]. Investigations of RTX PK in diffuse large B-cell lymphoma (DLBCL) showed a faster clearance (12.68 vs. 8.21 mL/h, *p* = 0.003) in males compared to females, with a significantly shorter terminal half-life (*t*^1/2^ = 24.7 vs. 30.7 days, *p* = 0.003) [323]. Correspondingly, poorer clinical outcome was observed in men, and the male gender is considered as an adverse prognostic factor in RTX therapies of DLBCL (event free survival 63% in women vs. 46% in men) [324] as well as NHL and CLL [325]. Given the significance of TMDD for RTX, differences in CD20 expression between genders may contribute to the observed results; however, the impact of gender on CD20 expression has not been reported (to our knowledge).

### 4.3. Race

Individuals of European descent constitute 77% of the United States population, and patients from minority ethnic groups are not well represented, historically, in clinical trials. After accounting for BW, race is seldom found to be a significant covariate for mAb PK [326,327]. Ling et al. evaluated the differences between Caucasian and Japanese subjects but found no significant differences in exposure following SC dosing of golimumab [71]. Similar results were found for omalizumab PK in Japanese and Caucasian populations [328,329]. Differences in gene/target expression [330], tumor burden [331], disease progression, FcγR polymorphism have been noted between different ethnic groups, but a clinically significant effect on mAb PK has not been recorded in the clinic. Nonetheless, the effects of race may be understudied, and long-term studies in diverse populations may be needed to evaluate appropriately the role of race on mAb PK [332].

### 4.4. Age

For most clinical trials, the adult patients enrolled have a broad range of age; however, age is rarely identified as a significant covariate on mAb PK parameters. Among approved mAbs, age was found to correlate with efalizumab clearance in psoriasis patients, but the effect was modest [326]. Age has been found to be a significant covariate for the rate of absorption (ka) for anti-interleukin receptor IL-4Rα (AMG 317) in healthy subjects [48], denosumab in women with osteoporosis [46] and in solid tumors [47], and for canakimumab in healthy patients [44] and patients with gouty arthritis [45].

Developmental differences between adults and children in body size, physiological maturation, disease activity, target expression, lymph flow rates, and ADA response may contribute to variability in mAb PK. Nine mAbs are approved in United States in pediatric populations. In many cases, the mAb dose applied to pediatric patients is derived using linear extrapolation of the adult regimen, as ethical and practical impediments limit pediatric clinical trials [333]. Consistent relationships between mAb PK parameters in pediatric relative to adult patients have not been found. For example, alemtuzumab clearance is faster in pediatric patients [334], but basiliximab clearance is slower in pediatric vs. adult renal transplant patients [335]. In most cases, a similar clearance has been found (e.g., gemtuzumab in acute myeloid leukemia [336], bevacizumab [337], IFX [338], cetuximab [339]). A higher absorption of mAb has been observed in younger subjects compared to adults [48]. Lowe et al. developed a model to predict omalizumab concentrations in patients 12–79 years of age. The model was able to correlate the suppression of free IgE concentration to improvement in clinical outcome [340]. In the geriatric patients, the physiological functions like lymph flow rate, target expression, vascular permeability, hormones may have undergo changes, but no significant findings have been observed in the clinic.

### 4.5. Albumin

Albumin, the most abundant protein in the plasma, is primarily produced by the liver and widely distributed between the intravascular and extravascular space. Albumin is routinely evaluated as a covariate for mAb PK parameters. Progressive loss of vital proteins during an inflammatory response may cause hypoalbuminemia and can be prognostic marker [341]. Albumin correlates with BW, and may be considered as a derived anthropometric parameter; however, its significance as a covariate may relate to the fact that albumin, like IgG, is protected from intracellular catabolism by FcRn. Albumin binds to FcRn non-cooperatively at a site distinct from IgG [342]. Although the impact of FcRn on IgG clearance is much greater than the impact of FcRn on the clearance of albumin, owing to the much higher concentration of albumin relative to IgG, it is estimated that 35-fold more albumin is salvaged by FcRn per unit time (i.e., relative to FcRn salvage of IgG) [343].

In clinical trials, serum albumin was identified as a significant covariate explaining IIV in clearance for IFX (in ulcerative colitis [320] and Crohn’s disease [344]), ustekinumab [279], and pertuzumab [345]. For other mAbs including trastuzumab [346] and golimumab (psoriatic arthritis [277] and ankylosing arthritis [276]), albumin was not found to be a statistically significant covariate for clearance. Serum albumin concentration was found to correlate inversely with IFX elimination in ulcerative colitis patients [320]. Fasanmade et al. evaluated serum albumin as a predictive factor in ulcerative colitis and inferred that the relationship between steady-state albumin concentration and IFX clearance might be explained by FcRn expression, efficiency, and/or activity [347]. Although the FcRn hypothesis has not been tested thoroughly, increased protein catabolism due to disease, increased renal excretion due to kidney dysfunction, decreased production due to liver dysfunction, and loss of protein into the gut due gastrointestinal pathology are additional mechanisms that may explain the observed results. In a preclinical study by Engler et al., urinary albumin excretion (UAE) rate was used as a covariate to explain the increase in mAb clearance observed in mouse model of diabetic nephropathy, greatly reducing residual variability [348]. Diabetic nephropathy may lead to damage of glomeruli, increasing porosity and increasing the filtration of albumin and IgG into the urine (discussed below).

## 5. Additional Factors Contributing to Inter-Individual Variability

### 5.1. Influence of Pathophysiological Elements of Disease

#### 5.1.1. Proteinuria and Renal Protein Catabolism

Antibodies are not efficiently eliminated via renal filtration and subsequent catabolism due their large size; hence, this pathway is considered inconsequential to mAb clearance [97]. Disease related damage to the kidneys caused by leucocyte infiltration and inflammatory mediators may increase the radius of glomerular pores and increase glomerular filtration of protein. Increased renal protein filtration is accompanied by increased proximal renal tubular protein catabolism. The kinetics of protein catabolism in patients have been found to correlate with proteinuria [349]. Renal impairment is commonly observed in diseases like diabetes mellitus, SLE, RA, sarcoidosis, multiple myeloma and CLL.

Around 40% of patients with diabetes mellitus develop diabetic nephropathy (DN) [350] resulting from progressive glomerular injury/sclerosis, loss of charge-dependent restriction of protein, and podocyte insufficiency. DN leads to a loss of glomerular size selectivity, which precipitates microalbuminuria and, as the condition advances, overt macroalbuminuria. Numerous clinical studies have reported severe IgG loss via urine in patients with DN. Several fold increases in urine IgG concentration were reported in Pima Indians with type 2 diabetes with microalbuminuria or macroalbuminuria [351]. A greater than 20% increase in ustekinumab clearance was observed in plaque psoriasis patients having diabetes compared to non-diabetic psoriasis patients [279]. Urinary IgG has been also suggested as a marker for proteinuria progression in type 2 diabetic patients [352]. Preclinical investigations carried out by Engler et al. reported a 1.8-fold increase in mAb clearance in a mouse model of streptozotocin-induced DN. Increases in mAb clearance were found to strongly correlate with urinary albumin excretion rate [348]. Similar results were obtained by Chadha and Morris with a 3.5-fold increase mAb clearance in the Zucker diabetic rat model [353].

Faster elimination of IgG in SLE patients has been reported [354,355], and a preclinical study carried out in mouse models with lupus-like autoimmune syndromes have reported several fold increases in IgG clearance [356]. Around 60–66% of patients with SLE develop lupus nephritis, where proteinuria can exceed 6 g/day [357]. Significant associations between belimumab clearance in SLE patients and proteinuria have been reported, but the available data are somewhat limited as clinical investigations of belimumab excluded patients with proteinuria >6 g/day [358]. In SLE patients with lupus nephritis, proteinuria can be severe, ranging from 6–30 g/day for many patients. A more direct clinical investigation evaluating effect of severe lupus nephritis on mAb PK has not yet been performed. Other mechanisms like saturation of FcRn due to increases endogenous IgG production, loss of mAb due to co-morbidities such as protein losing enteropathy, and disease-related changes in FcRn expression/function may also influence mAb PK.

Approximately 17% of patients with RA develop nephropathy with moderate proteinuria [359]. Although the clinical relevance of RA associated nephropathy on mAb PK has received little investigation, five-fold decreases in RTX concentrations were observed in RA patients with idiopathic membranous nephropathy with proteinuria vs. RTX concentrations found in RA patients without proteinuria [360]. RTX was recently introduced as a second in line treatment for pediatric nephrotic syndrome, and was found to have a very short half-life (less than a day). RTX failed to meet clinical efficacy end-points, possibly due to excessive loss of RTX via non-selective proteinuria [361]. Proteinuria is also frequently observed in other diseases including sarcoidosis (7–27%), CLL (42%), renal cell carcinoma, and multiple myeloma [362,363,364].

#### 5.1.2. Protein Losing Enteropathy

Protein losing enteropathy (PLE) is a co-morbidity characterized by a loss of gastrointestinal integrity due to mucosal disruption, lymphatic channel obstruction, or gut wall erosion/ulceration. PLE has been associated with inflammatory bowel disease, Crohn’s disease, ulcerative colitis, RA, gastric cancer, and SLE.

In preclinical investigations carried out in the Balthasar laboratory, the influence of PLE on mAb PK was quantified using a mouse model [365]. Compared to control, the area under the plasma concentration-time curve of 8C2, a model IgG1 mAb, was substantially reduced in PLE mice (1368 ± 255 vs. 594 ± 224 day·µg/mL, *p* = 0.001). Alpha-1 antitrypsin (A1AT), a protease inhibitor which is resistant to degradation by fecal enzymes, is considered a reliable disease biomarker to assess gastrointestinal loss of plasma proteins in human subjects [366]. In the preclinical mouse study referenced above, A1AT was measured and used as marker to predict mAb clearance. Using population PK modeling, a quantitative relationship was developed between A1AT clearance and 8C2 clearance [365].

Interestingly, the impact of PLE on mAb PK may explain the clinical observation that mAb clearance is significantly increased in patients with gastric cancer, as PLE is commonly observed as a co-morbidity for this disease. For example, in patients with advanced gastric/gastroesophageal junction cancer, the median values of trastuzumab AUC and Cmax were 30–40% lower than values found in patients with metastatic breast cancer [367]. Median bevacizumab clearance was increased by 50% in patients with in advanced gastric cancer when compared to bevacizumab clearance in patients with cancers (4.5 vs. 3 mL/day/kg, *p* < 0.05) [368]. Similar results were obtained for pertuzumab in HER-positive advanced gastric cancer where the trough concentration (Cmin) observed was 37% lower as compared to values found in patients with metastatic breast cancer [369]. Further clinical evaluation is needed to determine whether these observations relate to the development of PLE. The role of PLE is likely to significant, and underappreciated, in severe ulcerative colitis, where fecal loss of IFX has been detected in 66% of patients, and where the observed fecal loss of IFX was correlated to a lack of clinical response [370].

#### 5.1.3. Blood-Brain Barrier

Currently, mAbs are in clinical trials for Alzheimer’s disease (AD), Huntington’s disease, and Parkinson’s disease [371]. However, there is a significant blood-brain barrier for IgG mAb, and antibody concentrations in the brain are often reported to be 1:100–1:1000 of concentrations found in plasma. There is some debate and uncertainty regarding the mechanisms responsible for the relatively low concentrations of IgG in the brain. One hypothesis is that FcRn mediates ‘reverse transcytosis’ of IgG, actively effluxing IgG molecules from brain interstitial fluid across the brain vascular endothelium [372]. FcRn is highly expressed in the brain capillary endothelium and choroid plexus epithelium [373]. However, investigations in mouse FcRn knockout models have not shown a significant impact of FcRn on brain: blood or brain: plasma exposure ratios for model mAb [374,375]. Subsequent investigations conducted by Yip et al., which employed engineered mAb with low FcRn binding affinity, demonstrated similar data as found in the knockout studies, where FcRn affinity did not influence brain to plasma mAb exposure ratios [108]. On the other hand, some data have been published in support of the reverse transcytosis hypothesis [372,373,376,377].

There is little debate that tight junctions between brain vascular endothelial cells contribute to the blood–brain barrier, decreasing the efficiency of paracellular transport of macromolecules from plasma to brain interstitial fluid. Inflammatory processes are well known to increase vascular porosity, thereby facilitating the entry of immune cells at sites of inflammation. There are contradictory reports on the influence of inflammatory processes, including central nervous system (CNS) diseases such as AD, on the integrity of the blood-brain barrier [378,379], and the impact of CNS disease on mAb delivery to the brain has not been defined.

### 5.2. Influence of Co-Administered Drugs

#### 5.2.1. Saturation of Neonatal Fc Receptor

IVIG therapy has been in the clinic to treat autoimmune conditions since 1981, when Imbach and coworkers demonstrated the efficacy of IVIG for the treatment of immune thrombocytopenia [380]. Although many mechanisms may contribute to the effects of IVIG in autoimmunity [381], it is now clear that a substantial contribution relates to the influence of IVIG on FcRn-mediated transport of pathogenic IgG. As discussed above, FcRn protects IgG antibodies from intracellular catabolism; however, the transport mediated by FcRn is, of course, capacity-limited. IVIG therapy employs doses of up to 2 g/kg of pooled IgG, which leads to a substantial increase in IgG concentrations in plasma and in other biofluids. The increased IgG concentrations saturate FcRn, leading to a transient increase in IgG elimination, including the elimination of pathogenic IgG antibodies associated with humoral autoimmune conditions. Preclinical support for this mechanism was provided through a series of investigations in rodent models that showed that IVIG leads to a dose-dependent increase in the clearance of 7E3, a model anti-platelet IgG mAb, [101], and that IVIG treatment did not increase 7E3 clearance in FcRn knockout mice (supporting the hypothesis that IVIG effects on 7E3 PK were mediated by FcRn) [382]. Further work demonstrated that similar effects on mAb PK could be achieved by high dose administration of mAb (i.e., indicating that the PK effect was not related to non-IgG substances that are present in clinical preparations of IVIG) [383]. Given that this interaction is mediated by saturation of FcRn, which is a determinant of the PK of virtually all therapeutic IgG mAb, increased mAb clearance with concomitant IVIG therapy should be anticipated for all mAb.

#### 5.2.2. Alteration in Convective Transport and Tumor Uptake

Preclinical investigations have evaluated the effects of antibody and small molecule anti-angiogenic agents on the tumor uptake and distribution of tumor-specific mAb. Effects of anti-vascular endothelial growth factor (VEGF) therapy on the pharmacokinetics of T84.66, an anti-carcinoembryonic antigen (CEA) mAb, was examined in a CEA expressing colorectal xenograft mouse model. The results demonstrated a reduction in the area under the tumor concentration v. time curve of T84.66 in anti-VEGF treated, with no significant effects of anti-VEGF therapy on plasma PK or on non-tumor tissue PK [384]. Similar observations were made by Pastuskovas and co-workers; bevacizumab therapy was found to decrease tumor exposure of trastuzumab and isotype control mAbs in a human epidermal growth factor receptor (HER-2) expressing xenograft mouse model [385]. In another preclinical investigation, the effect of the small molecule anti-angiogenic agent sorafenib on T84.66 tumor disposition was evaluated. Sorafenib treatment decreased tumor microvessel density, decreased macromolecular extravasation in tumors, and decreased tumor exposure to T84.66 [386].

Numerous clinical trials have evaluated the efficacy of combination therapies involving anti-angiogenic agents and tumor-specific mAbs. Administration of sorafenib with IFX [387], bevacizumab [388], tigatuzumab [389], and ramucirumab [390] in different cancers failed to improve the primary efficacy endpoint, when compared to sorafenib or mAb monotherapy. However, some mAb-sorafenib combination therapies have shown clinical benefit [391,392,393]. Similarly, the addition of bevacizumab to trastuzumab therapy in HER-2 positive breast cancer patients did not improve overall survival [394]. Muselaers et al. used an imaging technique to show that 4-week sorafenib treatment (400 mg twice daily) in patients with renal cell carcinoma decreased microvessel density in tumor tissue and caused a 38.4% reduction in the uptake of 111In-girentuximab [395], in line with preclinical results [386].

#### 5.2.3. Decrease in Anti-Drug Antibody Response

Observations originating in the late 1990s have shown that co-administration of mAb with some immunosuppressive drugs decreases mAb clearance and decreases the development ADA (e.g., methotrexate [289], azathioprine [290], and mycophenolate mofetil [290,291]). Although there is a rather limited understanding of the mechanistic determinants of ADA development and immunogenicity, and there is also little mechanistic understanding of the effects of immunosuppressive therapy on ADA development, it is very well established that immunosuppressive agents decrease ADA development and, thus, impact mAb PK. As such, therapy with agents such as methotrexate, azathioprine, and mycophenolate mofetil may influence the IIV of mAb PK, and may warrant consideration within covariate analyses in population PK analyses.

#### 5.2.4. Alteration in Target Expression and Conformation

It is plausible that the administration of some therapeutic agents will alter the expression or confirmation of target proteins and, consequently, alter the target-mediated disposition or pharmacodynamics of mAb. Interestingly, the combination of anti-CD20 mAbs with ibrutinib, a kinase inhibitor, has been reported to lead to an antagonistic interaction, where ibrutinib downregulates CD20 expression (in vivo) and inhibits ADCC and antibody-dependent cellular phagocytic activity mediated by RTX, ofatumumab, and obinutuzumab [396,397,398]. Given that anti-CD20 mAb exhibit TMDD, it may be expected that downregulation of CD20 will impact mAb PK (e.g., decreasing clearance); however, the pharmacokinetic implications of ibrutinib on anti-CD20 mAb require clinical evaluation.

Statins (lipid lowering agents) are commonly administered for hypercholesterolemia and for the prevention of cardiovascular diseases. Statins are typically employed as a chronic therapy, and many patients are prescribed daily dosing. In an in vitro study conducted with freshly isolated B cells, statins were found to cause conformational changes to CD20 and it has been suggested that the effects of statins on CD20 and on cholesterol homeostasis may impair recognition of CD20 and decrease RTX-mediated CDC [399]. Additionally, statin therapy can lead to increased expression of proprotein convertase subtilisin/kexin type 9 (PCSK9), which is the pharmacological target for anti-PCSK9 antibodies such as evolocumab and alirocumab. Statin therapy has been shown to increase the clearance of anti-PCSK9 mAb, leading to a 20% decrease in Cmax and AUC of evolocumab, presumably by increasing the extent of target-mediated evolocumab elimination [400]. Similarly, statin therapy has been shown to lead to a 40% decrease in exposure of alirocumab [401].

Co-administration of drugs targeting the same antigen may lead to alterations in mAb distribution and elimination. Recently, Cilliers et al. showed that co-administration of trastuzumab with ado-trastuzumab emtansine (T-DM1) improved the tumor distribution of T-DM1 by competing with T-DM1 and allowing better penetration of T-DM1 (by overcoming binding site barrier) within tumor tissue [402]. In another example, the co-administration of two anti-CD20 drugs, inotuzumab ozogamicin (INO, anti-CD22 mAb) and RTX (anti-CD20), was simulated with the use of a mathematical model. The model predicted that RTX would decrease the target-mediated non-linear clearance of INO by depleting the B cells, thus explaining the 14% decrease in INO clearance that had been observed clinically [403].

## 6. Summary and Future Prospects

In the above sections, we compiled the critical mechanisms of antibody absorption, distribution and elimination to facilitate a comprehensive discussion on the variability in the determinants of antibody disposition. We discussed evidence, possible hypotheses and examples from in vitro, preclinical and clinical studies, identifying sources of IIV that may warrant consideration in population PK analysis. The biological relevance of several of the commonly considered covariates was highlighted. In addition, we assessed the influence of factors like disease and co-administered drug on IIV. The review addresses the potential sources of IIV and strives to give a mechanistic understanding of how these factors may contribute to the variability observed.

Over the past two decades, there has been a great increase in understanding in the mechanistic determinants of the PK of monoclonal antibody drugs. The significance of, and the determinants of, target-mediated distribution and elimination, FcRn transport, interaction with FcγR, and the role of ADA are more fully appreciated within the pharmaceutical industry. This improved mechanistic understanding is enabling the development and facilitating the testing of hypotheses to explain the high degree of inter-individual variability that has been observed for mAb PK. It is anticipated that the combined application of population PK modeling, detailed mechanistic explorations with preclinical models, focused clinical investigations, and mechanistic mathematical modeling will pave the way for personalized mAb therapy, potentially allowing increased efficacy, while decreasing toxicity and cost.

## Figures and Tables

**Table 1:** Results from studies investigating the role of the lymphatic system in the subcutaneous absorption of proteins.

Species	Model Protein	Molecular Weight (kDa)	Site of Injection	% Dose Recovery in Lymph	Selected Lymph Duct/Node	Does the Lymphatic System Contribute to SC Absorption?	Ref.
Sheep	5-fluoro-2′deoxyuridine	0.246	Lower part of right hind leg	4.0 ± 1.5	Efferent duct of the popliteal lymph node	Yes	[17]
Sheep	Insulin	5.80	Interdigital space of hind leg	17.3 ± 1	Efferent duct of the popliteal lymph node	Yes	[18]
Sheep	Inulin	5.20	Lower part of right hind leg	21.0 ± 7.1	Efferent duct of the popliteal lymph node	Yes	[17]
Sheep	Cytochrome c	12.3	Lower part of right hind leg	38.6 ± 6.7	Efferent duct of the popliteal lymph node	Yes	[17]
Sheep	Recombinant methionyl human leptin	16.2	Interdigital space of hind leg	34.4 ± 9.7	Efferent duct of the popliteal lymph node	Yes	[19]
Sheep	Human recombinant interferon-2α	19.0	Lower part of right hind leg	59.5 ± 7.1	Efferent duct of the popliteal lymph node	Yes	[17]
Sheep	Human growth Hormone	22.0	Interdigital space of hind leg	61.7 ± 8.5	Efferent duct of the popliteal lymph node	Yes	[20]
Sheep	Recombinant human epoetin-α	30.4	Interdigital space of hind leg	83.9 ± 6.6	Efferent duct of the popliteal lymph node	Yes	[21]
Sheep	Darbepoetin- α	37.0	Interdigital space of hind leg	90.2 ± 4.4	Efferent duct of the popliteal lymph node	Yes	[22]
Dog	PEGylated recombinant human erythropoietin α	60.4	Lower left region of hind leg	20	Thoracic lymph duct	Yes	[23]
Dog	PEGylated neuromedin-U receptor agonist MRL-1	46.0	Popliteal region of hind limb	72.9	Thoracic lymph duct	Yes	[24]
Rabbit	Interferon- α_2_	19.2	Hind leg	0.10 ± 0.06	Thoracic lymph duct	No	[26]
Rat	Recombinant human tumor necrosis factor-α	45.0	Back region	4.7 ± 3.4	Thoracic lymph duct	No	[27]
Rat	Bovine insulin	5.60	Lateral side of thigh	0.072 ± 0.0016	Thoracic lymph duct	No	[28]
Rat	Recombinant human erythropoietin α	30.4	Lateral side of thigh	1.44 ± 0.26	Thoracic lymph duct	No	[28]
Rat	Bovine serum albumin	66.0	Lateral side of thigh	2.15 ± 1.08	Thoracic lymph duct	No	[28]
Rat	PEGylated poly-L-lysine Lys_16_ (PEG_2000_)_32_	68.0	Lower right hind leg	29	Thoracic lymph duct	Yes	[29]
Rat	PEGylated recombinant human erythropoietin α	60.4	Lower left hind leg	23.8 ± 1.08	Thoracic lymph duct	Yes	[23]
Rat	PEGylated neuromedin-U receptor agonist MRL-1	46.0	Lower left hind leg	26.7 ± 9.0	Thoracic lymph duct	Yes	[24]
Rat	Trastuzumab	149	Inner left hind leg	26.7 ± 10.4	Thoracic lymph duct	Yes	[30]

**Table 2:** Human and mouse family of Fc gamma receptors.

**Human Fc Gamma Receptors**											
**Name**		**FcγRI (CD 64)**	**FcγRIIA (CD 32A)**		**FcγRIIB (CD 32B)**		**FcγRIIC (CD 32C)**		**FcγRIIIA (CD 16A)**		**FcγRIIIB (CD 16B)**
Gene [117]		*FCGRT1A*	*FCGRT2A*		*FCGRT2B*		*FCGRT2C*		*FCGRT3A*		*FCGRT3B*
Alleles [117]		-	H131	R131	I232	T232	Q13	Stop13	V158	F158	NA1
											NA2
											SH
Affinity for Ligand [117] (M^−1^)	IgG1	6 × 10^7^	5 × 10^6^	3 × 10^6^	3 × 10^8^	*ND*	1 × 10^5^	*NB*	2 × 10^5^	1 × 10^5^	2 × 10^5^
	IgG2	*NB*	4 × 10^5^	1 × 10^5^	1 × 10^5^	*ND*	2 × 10^4^	*NB*	7 × 10^4^	3 × 10^4^	*NB*
	IgG3	6 × 10^7^	9 × 10^5^	9 × 10^5^	9 × 10^5^	*ND*	2 × 10^5^	*NB*	1 × 10^7^	8 × 10^6^	1 × 10^6^
	IgG4	3 × 10^7^	2 × 10^5^	2 × 10^5^	2 × 10^5^	*ND*	2 × 10^5^	*NB*	2 × 10^5^	2 × 10^5^	*NB*
Cell Distribution [117,118]		Macrophages	Macrophages		Macrophages		Macrophages		Macrophages		Neutrophils
		Eosinophils	Eosinophils		Eosinophils		Neutrophils		Dendritic cells		
		Dendritic cells	Dendritic cells		Dendritic cells		NK cells		Basophils		
		Neutrophils	Neutrophils		Neutrophils				Mast cells		
			Mast cells		Mast cells				NK cells		
			Platelets		B cells						
Class		Activation	Activation		Inhibition		Activation		Activation		Decoy
											Activation (not clear)
Function [119,120]		Effector cell activation	Effector cell activation		Inhibition of effector activity		Co-activation receptor for FcγRIIIA, ADCC		Effector cell activation		Unknown
		Phagocytosis	Phagocytosis						Phagocytosis		
			Degranulation						ADCC		
			ADCC								
**Mouse Fc Gamma Receptors**											
**Name**		**FcγRI (CD 64)**	**FcγRIIB (CD 32B)**				**FcγRIII (CD 16)**		**FcγRIV (CD 16-2)**		
Affinity for Ligand [118] (M^−1^)	IgG1	*NB*	3.3 × 10^6^				0.3 × 10^6^		*NB*		
	IgG2a	1.6 × 10^8^	0.4 × 10^6^				0.7 × 10^6^		2.9 × 10^7^		
	IgG2b	*NB*	2.2 × 10^6^				0.6 × 10^6^		1.7 × 10^7^		
Cell Distribution [118]		Monocytes	B cells				Monocytes		Monocytes		
		Macrophages	Dendritic cells				Macrophages		Macrophages		
		Dendritic cells					Neutrophils		Neutrophils		
							Dendritic cells		Dendritic cells		
							NK cells				
Class		Activation	Inhibition				Activation		Activation		

Abbreviations: FcγR Fc gamma receptors, CD Cluster of Differentiation, IgG Immunoglobulin, NK Natural Killer cells, *ND* Not Determined, *NB* No Binding, ADCC Antibody Dependent Cell Cytotoxicity.

**Table 3:** Examples of targets that are highly variable in expression.

Target	Disease	Number of Patients	Target Expression	Unit	Fold Range in Expression	Approved mAb	Ref.
CD-20	Chronic lymphocytic leukemia	31	2737–115623	MESF	42	Rituximab	[208]
						Ofatumumab	
CD-20	Diffuse large B-cell lymphomas	64	3549–679577	MESF	191	Rituximab	[208]
						Ibritumomab	
CD-20	Follicular Lymphoma	56	8460–445755	MESF	52	tiuxetan	[208]
CD-20	Mantle Cell Lymphoma	34	8826–423799	MESF	48	Tositumomab	[208]
CD-20	Marginal Zone Lymphoma	18	3615–207034	MESF	57	(I-131)	[208]
CD-52	Chronic lymphocytic leukemia	5	371303 ± 117212	Receptor Number	-	Alemtuzumab	[209]
TNF-α	Rheumatoid Arthritis	327	0.92–9.68	pg/Ml	10.5	Adalimumab	[198]
						Certolizumab	
						Golimumab	
						Infliximab	
CD25	Kidney Transplantation	14	57.1 ± 12.7	Mean % of CD25 + within CD4 + T cells	-	Basiliximab	[210]
IgE	Asthma	245	51–1692	ng/mL	33	Omalizumab	[211]
VEGF	Advanced Breast Cancer	56	12.5–445 (plasma)	pg/mL	35	Bevacizumab	[212]
EGFR	Colorectal Adenocarcinoma	143	1 (10%)	IHC score (% of patient)	-	Cetuximab	[213]
			2 (32%)			Panitumumab	
			3 (55%)				
HER-2	Breast Cancer	47	1 (49%)	IHC score (% of patient)	-	Trastuzumab	[214]
			2 (6%)			Pertuzumab	
			3 (55%)				

**Abbreviations:** MESF Molecules of equivalent soluble fluorochrome. CD Cluster of Differentiation, TNF α Tumor Necrosis Factor alpha, IgGE Immunoglobulin E, VEGF Vascular Endothelial Growth Factor, EGFR Epidermal Growth Factor Receptor, HER 2 Human Epidermal Growth, Receptor, IHC score Immunohistochemistry score.

**Table 4:** Impact of anti-drug antibody formation on mAb pharmacokinetics.

mAb	Type	Antigen	Route	Disease	% Immunogenicity (*n* = Total Number of Patients)	Impact on PK	Ref.
Infliximab	Chimeric IgG1k	TNF-α	IV infusion	AS	25% (*n* = 8)	NQ	[272]
				RA	33% (*n* = 143)	NQ	[273]
				Psoriasis	19–22% (*n* = 264)	NQ	[293]
				CD	61% (*n* = 125)	NQ	[294]
				IBD	15% (*n* = 33)	2.7 fold ↑ in CL; 34% ↓ in *t*_1/2_	[275]
Adalimumab	Human IgG1k	TNF-α	IV	RA	28% (*n* = 272)	1.4 fold ↓ in mAb, median concentration	[295]
				Psoriasis	49% (*n* = 80)	Significant ↓ in mAb, Cmin	[296]
				CD	13–18% (*n* = 65 to 96)	4–5.5 fold ↑ in CL	[297,298]
Natalizumab	Humanized IgG4k	α4-Integrin	IV infusion	MS	9–82% (*n* = 2195)	3 fold ↑ in CL	[299]
				CD	5–10% (*n* = 1414)	NQ	[299]
Golimumab	Human IgG1k	TNF-α	SC	PA	2.90% (*n* = 337)	Antibody to mAb significant covariate on CL/F	[277]
				AS	3.10% (*n* = 312)	36% ↑ in median CL/F	[276]
Ustekinumab	Human IgG1k	IL-12/IL-23	SC	Psoriasis	3.20% (*n* = 1937)	35.5% ↑ in median CL/F	[279]
				PA	9.20% (*n* = 130)	42% ↑ in median CL/F	[278]
Anti-IL-1β	Humanized IgG4	IL-1β	SC	T2DM	36.7% (*n* = 79)		
			IV	RA	2.1% (*n* = 96)	37.6% ↑ in CL	[280]
Daclizumab	Humanized IgG1	IL-2 Receptor α	SC	Remitting relapsing MS	0.80% (*n* = 17139)	19% ↑ in median CL	[281]
Amatuximab	Chimeric IgG1k	Mesothelin	IV infusion	Unresectable malignant pleural mesothelioma	24.60% (*n* = 199)	1.5 fold ↑ in CL	[282]
Atezolizumab	Humanized IgG1	PD-L1	IV infusion	Metastatic Urothelial Carcinoma	31.70% (*n* = 139)	16% ↑ in median CL	[283]
Benralizumab	Humanized IgG1	IL-5 receptor α	SC	Asthma	9.50% (*n* = 200)	4.6 fold ↑ in median CL	[284]

Abbreviations: IgG-Immunoglobulin, TNF α-Tumor Necrosis Factor Alpha, IL-Interleukin, IV-Intravenous, SC-Subcutaneous, AS-Ankylosing Spondylitis, RA-Rheumatoid Arthritis, CD-Crohn’s Disease, IBD-Inflammatory Bowel Disease, MS-Multiple Sclerosis, PA-Psoriatic Arthritis, T2DM-Type II Diabetes Mellitus, NQ-Not quantified, CL-Clearance, F-Bioavailability, *t*_1/2_-half life, Cmin-mAb trough concentration.
